# Sourcing brain histone modification data and development of algorithm for identification of hypersensitive sites

**DOI:** 10.1186/1471-2202-16-S1-P188

**Published:** 2015-12-18

**Authors:** Victor Chukwudi Osamor

**Affiliations:** 1 Department of Computer and Information Sciences, Covenant University, P.M.B 1023, Ota, Ogun State, Nigeria; 2 Institute of Informatics, University of Warsaw, ul Banacha 2, 02-097, Warsaw, Poland

## Background

The source of data for computational analysis goes a long way to determine the quality of data used and the computed result. This is due to the differences in the avalanche of protocols applicable in experimental data generation, professional skills, endurance, diligence and consideration to details by investigators. The relevance of identification of DNase hypersensitive sites [1] gives a clue to the role of genes based on the transcription binding properties of various regions. The aim of this study is to conduct a comparative assessment of the ready availability of brain histone modification data and propose an algorithm for the use of such data for transcription factor dimer prediction.

## Methods

We carried out an extensive analysis on Encyclopedia of DNA Elements (ENCODE) [2] to determine the easiest way of accessing histone modification (HM) data [3]. We compared the process of entering the web address of http://genome.ucsc.edu/ to invoke the genome browser by a click → Select the human genome and click submit→Scrolling downwards till "Regulation" which is a blue horizontal band → finding and clicking "ENCODE Histone Modification" → Selecting the source of desired histone among four different sources and finally downloading from huge array of scrolling pages of data to locate your data of interest. This is quite cumbersome compared to a second method goggling "ENCODE Data Matrix" and select ChIP-seq option to see all the data content arranged neatly in form of a matrix. Comparatively, we considered 12 HMs in all for both Tier 1 and brain cell lines. Using Tier 1 (GM12878, H1hESC, K562) cell lines, we compared their availability with H1-Neurons and other brain cell-type viz Glioba, Medullo NH-A, Hac, Be2c and Sknshra as depicted in Table 1 with symbol of + representing present and - representing absent.

**Table 1:** Comparative histone modification analysis of Tier 1 (GM12878, H1hESC, K562) with others consisting of brain cell line and neuron.

	H2AFZ	H3K27ac	H3K27me3	H3K36me3	H3K4me1	H3K4me2	H3K4me3	H3K79me2	H3K9ac	H3K9me1	H3K9me3	H4K20me1	
GM12878	+	+	+	+	+	+	+	+	+	-	+	+	91.67%

HI-hESC	+	+	+	+	+	+	+	+	+	-	+	+	91.67%

K562	+	+	+	+	+	+	+	+	+	+	+	+	100.00%

H1-Neurons	-	-	-	-	-	-	-	-	-	-	-	-	0%

Glioba	-	-	-	-	-	-	-	-	-	-	-	-	0%

Medullo	-	-	-	-	-	-	-	-	-	-	-	-	0%

NH-A	+	+	+	+	+	+	+	+	+	-	+	+	91.67%

Hac	-	-	-	-	-	-	+	-	-	-	-	-	0%

Be2c	-	-	-	-	-	-	+	-	-	-	-	-	8.33%

Sknshra	-	-	+	+	-	-	+	-	-	-	-	-	25%


Symbol + indicates present and - indicates absent.

## Results

The HM of cell line in Tier 1 cell type is available for almost all considered available HMs while that of brain cells and H1-neurons were a either totally absent or scarcely available. In addition, Nh-A appears to be the only exceptional case where the availability is equal to the Tier 1 histone modification. We there evolved an algorithm that identifies hypersensitive sites from these data in the following way: 1. Run HM bam files on Model-based Analysis of ChIP-Seq(MACS) using non-modal, no lambda [4] parameter setting 2. If single HM, no merge is required then go to item 4. 3. If merge is required for a combination of HM peaks then (a) Append (b) Sort and (c) apply merge option 4. Prepare the various threshold to be clustered by a dimer prediction algorithm [5].

## Conclusions

A deluge of data can sometimes be bewildering especially when searching for a cell line of interest in a large database but we can arrest the difficulties following the proposed technique. The scarce presence of HM for brain cells calls for more attention on for the choice of such cell type for further investigation.
